# Innate Lymphoid Cells in Intestinal Inflammation

**DOI:** 10.3389/fimmu.2017.01296

**Published:** 2017-10-13

**Authors:** Alessandra Geremia, Carolina V. Arancibia-Cárcamo

**Affiliations:** ^1^Translational Gastroenterology Unit, Nuffield Department of Experimental Medicine, University of Oxford, Oxford, United Kingdom

**Keywords:** innate lymphocytes, epithelial barrier integrity, mucosal immune response, microbial flora, cytokines, inflammatory bowel disease, cancer

## Abstract

Inflammatory bowel disease (IBD) is a chronic inflammatory disorder of the intestine that encompasses Crohn’s disease (CD) and ulcerative colitis. The cause of IBD is unknown, but the evidence suggests that an aberrant immune response toward the commensal bacterial flora is responsible for disease in genetically susceptible individuals. Results from animal models of colitis and human studies indicate a role for innate lymphoid cells (ILC) in the pathogenesis of chronic intestinal inflammation in IBD. ILC are a population of lymphocytes that are enriched at mucosal sites, where they play a protective role against pathogens including extracellular bacteria, helminthes, and viruses. ILC lack an antigen-specific receptor, but can respond to environmental stress signals contributing to the rapid orchestration of an early immune response. Several subsets of ILC reflecting functional characteristics of T helper subsets have been described. ILC1 express the transcription factor T-bet and are characterized by secretion of IFNγ, ILC2 are GATA3^+^ and secrete IL5 and IL13 and ILC3 depend on expression of RORγt and secrete IL17 and IL22. However, ILC retain a degree of plasticity depending on exposure to cytokines and environmental factors. IL23 responsive ILC have been implicated in the pathogenesis of colitis in several innate murine models through the production of IL17, IFNγ, and GM-CSF. We have previously identified IL23 responsive ILC in the human intestine and found that they accumulate in the inflamed colon and small bowel of patients with CD. Other studies have confirmed accumulation of ILC in CD with increased frequencies of IFNγ-secreting ILC1 in both the intestinal lamina propria and the epithelium. Moreover, IL23 driven IL22 producing ILC have been shown to drive bacteria-induced colitis-associated cancer in mice. Interestingly, our data show increased ILC accumulation in patients with IBD and primary sclerosing cholangitis, who carry an increased risk of developing colorectal cancer. ILC may play an important amplifying role in IBD and IBD-associated cancer, through secretion of inflammatory cytokines and interaction with other immune and non-immune cells. Here, we will review the evidence indicating a role for ILC in the pathogenesis of chronic intestinal inflammation.

## Introduction

Innate lymphoid cells (ILC) belong to a family of innate immune cells that share similarities with the phenotype and functions of T lymphocytes. They present classical lymphocyte morphology, but lack a rearranged antigen-specific receptor. ILC do not express T, B, or myeloid cell markers and, therefore, they are lineage negative (Lin^−^), but express the IL2 receptor (CD25) and the IL7 receptor (IL7R or CD127) (1). ILC also include cytotoxic NK cells and lymphoid tissue inducer (LTi) cells that contribute to lymph node organogenesis during embryonic development (2).

**Figure 1:**
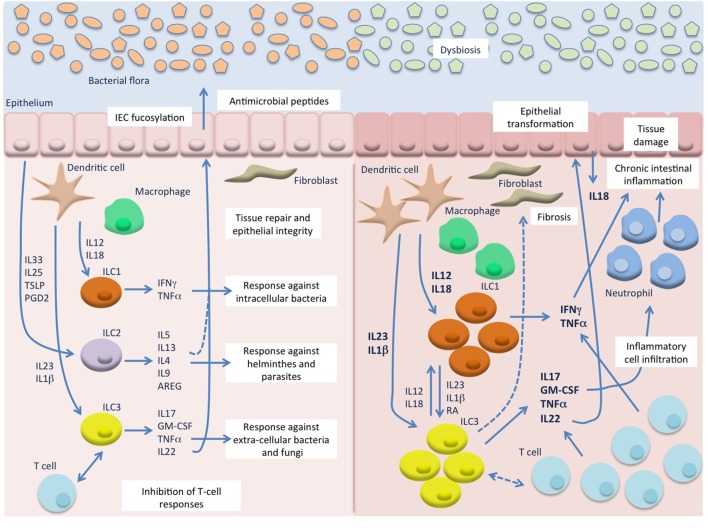
Innate lymphoid cells (ILC) in intestinal homeostasis and chronic inflammation. In the healthy mucosa (**left**) ILC contribute to the maintenance of intestinal homeostasis through induction of protective immune responses against pathogens and promotion of tissue integrity. In response to microbial stimuli, innate cells, such as dendritic cells (DC) and macrophages, secrete IL12 and IL18 that stimulate ILC1 responses against intracellular pathogens, and IL23 and IL1β that induce ILC3 responses against extracellular bacteria and fungi. On the other hand, epithelial derived factors, such as IL33, IL25, TSLP, and prostaglandin D2 (PGD2), induce secretion of type 2 cytokines from ILC2 that contribute to the expulsion of helminthes. ILC3 and possibly ILC2 also promote epithelial integrity and tissue repair, particularly through IL22 production, and can inhibit T cell activation and proliferation. During chronic intestinal inflammation, such as Crohn’s disease (CD) (**right**), in the presence of an altered bacterial flora, excessive levels of IL12, IL18, IL23, and IL1β are secreted by inflammatory DC and macrophages and lead to accumulation and activation of ILC1 and ILC3 that secrete high amount of type 1 and type 17 cytokines. These, also produced by accumulating T cells, induce chemotaxis of more inflammatory cells, such as neutrophils, and result in chronic inflammation and tissue damage. Besides the production of cytokines, ILC may also interact with immune (T cells) and non-immune cells (epithelial cells and fibroblasts) leading to secretion of more inflammatory mediators, such as IL18 from the epithelium, and to tissue reorganization (epithelial proliferation and fibrosis). A possible role for IL22 in epithelial transformation in colitis-associated cancer has also emerged from murine studies.

Innate lymphoid cells are crucial players mediating immunity against pathogens and maintenance of tissue homeostasis. They bridge the innate and adaptive immune systems, sense environmental changes, and are able to mediate these processes mainly by secreting and responding to a finely tuned cytokine crosstalk between various cell types. ILC are able to swiftly react to microbial and inflammatory challenges with cytokine production, limiting pathogen spread, and tissue injury. They are strategically located at sites where there is the highest exposure to the outside world and infections are more likely to first arise, such as the intestinal mucosa, the skin, and the lungs. They respond to environmental stress signals contributing to the rapid orchestration of an early immune response to pathogens, such as extracellular bacteria, helminthes, and viruses (Figure 1). However, recent evidence suggests that ILC are also involved in the pathogenesis of chronic inflammatory disorders, including allergic reactions, inflammatory bowel disease (IBD) and cancer (1).

Although it has been reported that human ILC express toll-like receptors (3), they mostly respond to cytokines by making more cytokines. Unlike innate cells that respond directly to the pathogens and adaptive cells that respond through more specific antigen recognition, ILC act like messengers that in response to innate cell produced cytokines secrete more cytokines to orchestrate the local immune response. A small population, through an impressive per cell ability to release cytokines, can have an important amplifying role and the combination of different cytokines can lead to a dramatic shift in responses. ILC also go further by interacting with other cells, such as non-immune cells like epithelial and stromal cells, and may induce tissue reorganization. Moreover, they can influence T cell responses by promoting T helper (Th) cell differentiation and effector functions, have been shown to express MHCII and are able to present antigen to Th cells and interact with regulatory T cells (Tregs) (4–7) (Figure 1).

Innate lymphoid cells arise from the common lymphoid progenitor and their development is dependent on the transcription factors Id2, the common γ-chain, GATA3, and PLZF (8–10). Three main subsets of ILC have been described reflecting functional characteristics of the Th subsets (2). Hence, like Th1, ILC1 express the transcription factor T-bet and are characterized by secretion of IFNγ and TNFα and are involved in the immune response against intracellular pathogens. ILC2, mirroring Th2 cells, express GATA3, secrete IL4, IL5, IL9, and IL13 and are involved in protection against helminthes and in allergic reactions. Finally, ILC3 depend on expression of RORγt and secrete IL17, IL22, GM-CSF, and TNFα, resembling their Th17 counterpart, and participate in the response against extracellular pathogens at mucosal sites (Figure 1).

However, it is becoming apparent that, similarly to what is observed for Th subsets, ILC retain a degree of plasticity depending on exposure to environmental stimuli, such as cytokines or metabolites. It has been shown that ILC1 can differentiate into ILC3 in presence of IL1β and IL23, an effect that is enhanced by the vitamin A metabolite retinoic acid (RA) (11), and to ILC2 in the presence of IL4 (12). On the other hand, ILC3 when exposed to IL12, IL18, and/or IL1β up-regulate T-bet and can therefore express IFNγ, while repressing RORγt and the secretion of IL17 and IL22 (13). In mice, a gradient of expression of RORγt and T-bet by ILC3 is associated with functional plasticity (14). In particular, NKp46^−^ ILC3 can develop into NKp46^+^ “ex-ILC3” through induction of T-bet and repression of RORγt. The “ex-ILC3” down-regulate the IL23 receptor, up-regulate the IL12 receptor, and mainly produce IFNγ in response to IL12. Similarly ILC2 can also trans-differentiate into ILC1 after exposure to IL12 and IL18 (12, 15).

Recent studies have particularly challenged the existence of a defined ILC1 subset, distinct from conventional NK cells, and other ILC subsets. Simoni *et al*. did not find human ILC1 in various healthy and disease-derived tissues using CyTOF mass-cytometry to assess the expression of an extensive range of cell surface and intracellular parameters. The t-SNE analysis showed clear clustering of ILC2, ILC3, and conventional NK cells, but was unable to identify a distinct subset of ILC1 that appeared instead to be scattered throughout other clusters (16). Furthermore, transcriptomic analysis of murine ILC and NK cell populations has shown overlapping profiles for ILC1 cells, NKp46^+^ “ex-ILC3” cells, and NK cells (17). On the other hand, in a human study using single cell RNA sequencing of tonsil derived CD127^+^ ILC and NK cells, unbiased clustering of cellular transcriptomes revealed four distinct cell populations, corresponding to ILC1, ILC2, ILC3, and NK cells (18). These observations suggest that a dogmatic classification of ILC in rigidly defined subtypes is likely to represent an over-simplification of the real scenario. It appears more plausible to conceive that ILC, like their T cell counterpart, can exert flexible and adaptable functional properties in response to the evolving environment at mucosal sites.

ILC are key contributors to the already complex immune system playing a critical role in health and disease. In this review, we focus on the role of non-cytotoxic or helper-like ILC (called ILC from now on for simplicity) in intestinal homeostasis and inflammation.

## ILC in Intestinal Homeostasis

The maintenance and regulation of the intestinal microenvironment to keep a stable and functional healthy gut are not a small task and involve constant tweaking of physiological, biochemical, and immune pathways. The intestinal environment has been described as the Wild West of the immune system and represents the epitome of the complexity of immune organization. In this environment, several factors, such as commensal microbes, foods, and metabolites, can modify the immunological balance of the region and the gut needs to distinguish unharmful from pernicious agents. The process of homeostasis in the intestine is not a passive process, but it actually involves several active mechanisms on the part of local cells especially from ILC.

The distribution of ILC varies across the different parts of the human gastrointestinal (GI) tract with a progressive increase of total ILC from the proximal to the distal intestine (19). Moreover, distinct subsets are represented at different sites. Indeed, ILC1 are enriched in the upper GI tract, while ILC2 are only present at very low frequencies in the entire intestine, and ILC3 increase toward the colon and this correlates with higher distal expression of IL7. Interestingly, the functional properties of these cells were also found to differ across different parts of the intestine, with highest secretion of IL22 being observed in the ileum and in the esophagus (19).

ILC play a role in the maintenance of intestinal homeostasis by contributing to the protective immune response toward intestinal pathogens, as it has emerged by murine studies. In particular, T-bet expressing ILC participate in the defense against *Helicobacter typhlonius*, a commensal in the murine microbiota that closely resembles *Helicobacter pylori*, the frequent colonizer of the human stomach associated with gastritis, peptic ulcer, and gastric cancer. Mice lacking T-bet in the innate immune compartment develop colitis spontaneously triggered by *H. typhlonius* through induction of TNFα secretion by dendritic cells (DC). In turn, TNFα synergizes with IL23 to induce IL17 secretion by ILC in this animal model (20). These findings are particularly interesting in light of the high representation of ILC1 in the human upper GI tract where they may be involved in the inflammatory and pro-carcinogenic responses induced by *H. pylori*. ILC1 were also shown to contribute to protection against intracellular pathogens, such as *Salmonella enterica*. During *Salmonella* infection T-bet expressing ILC (including ILC1 and “ex-ILC3”) are the main source of IFNγ, which drives the secretion of mucus-forming glycoproteins required to protect the epithelial barrier (14). Similarly, ILC1 are the main producers of both IFNγ and TNFα during *Toxoplasma gondii* infection and T-bet deficient mice fail to control parasite replication (21).

On the other hand, ILC2 were found to play an important role in mounting protective innate responses against parasites and helminthes through induction of eosinophilia and goblet cell hyperplasia. In particular, IL25 and IL33 responsive ILC2 are critical for *Nippostrongylus brasiliensis* expulsion in mice that lack adaptive immune cells (6, 22, 23). As for all other ILC, ILC2 development depends on the common γ-chain cytokine receptor, IL7 and the transcription factor Id2 and GATA3 (24, 25), and they also require RORα (26, 27) and TCF-1 (28). ILC2 respond to stimulation with epithelial derived cytokines, such as IL33, IL25, and TSLP, but also eicosanoids, such as prostaglandin D2 (PGD2) (29, 30) and leukotriene D4 (31), and secrete type 2 cytokines, mainly IL5 and IL13, but also IL4, IL9 (31–34), and the epidermal growth family member amphiregulin that is responsible for lung epithelial repair after murine infection with the H1N1 influenza virus (35).

Human ILC2 have been identified in the peripheral blood and in fetal and adult tissues, including the gut, even if at low frequencies (19, 36). These cells express the IL33R (ST2) and the IL25R, and are positive for the chemoattractant receptor-homologous molecule expressed on Th2 lymphocytes (CRTH2) (36). CRTH2 is a G-protein coupled receptor for PGD2, which is released by activated mast cells during allergic reactions. PGD2 binding to CRTH2 has been shown to induce ILC2 migration, production of type 2 cytokines, and up-regulation of IL33R and IL25R possibly creating a positive feedback loop that amplifies type 2 responses during allergy (30).

Three independent groups initially identified ILC2 in mice and applied different denominations to the groups, “natural helper cells,” “nuocytes,” and “innate helper 2 cells” (6, 22, 23). “Natural helper cells” were identified in lymphoid clusters associated with the adipose tissue in the peritoneal cavity. Interestingly, similar fat-associate lymphoid clusters (FALC) were also found in the human mesentery. These cells are Lin^−^, but express c-Kit, Sca-1, the IL7R and the IL33R, produce large amounts of Th2 cytokines, such as IL5 and IL13 and also induce B cell proliferation. After helminthic infection and in response to IL33, FALC Lin^−^c-Kit^+^Sca-1^+^ cells produce large amounts of IL13, which leads to goblet cell hyperplasia and contribute to the expulsion of helminthes. The location of these cells in the visceral fat, and not at the classical barrier surface, is interesting. Recent studies indicate that ILC2 play a role in adipose tissue deposition and metabolism. In fact, they were shown to promote weight loss and glucose tolerance, through induction of caloric expenditure and browning of the adipose tissue (37–40). “Nuocytes” were identified in the IL13 reporter mice as non-B, non-T lymphocytes that expand *in vivo* in response to IL25 and IL33, and mediate the early protective immune response to helminthes through secretion of IL13. Similarly, “innate helper 2 cells” were identified as Lin^−^, IL25, and IL33 responsive cells in IL4 and IL13 reporter mice. These cells are widely distributed and expand after helminthic infection and secrete IL13 resulting in worm clearance. More recently, it has been shown that IL33-mediated induction of IL13 by ILC2 is necessary to mediate protection against helminthes (41).

Around the same time, an IL25 responsive Lin^−^ multipotent progenitor precursor called MPP^type2^ was also identified in the gut-associated lymphoid tissue (GALT). These cells are also able to elicit protective type 2 responses during parasitic infection (42). Compared with the other three populations, however, MPP^type2^ are Id2 independent, show a distinct transcriptional profile and are able to differentiate into multiple monocyte/macrophage and granulocyte populations and are therefore classically not included in the ILC family (43).

ILC2 were shown to be critical for epithelial repair in the lung following influenza virus infection through secretion of amphiregulin (35). Murine gut-associated ILC2 can also secrete amphiregulin in response to IL33 and were shown to have a protective role in the dextran sodium sulfate model of intestinal damage and inflammation (44). These data suggest that intestinal ILC2 may play a role in the maintenance of epithelial integrity in the gut (Figure 1).

Together with fetal LTi cells, ILC3 belong to the Group 3 of ILC. Both types of cells depend on RORγt and IL7 for their development and function (2). The classical Group 3 LTi, in conjunction with stromal cells, are responsible for the induction of secondary lymphoid tissues during embryonic development (45). On the other hand, ILC3 expand postnatally in response to microbiota derived signals (46–48). Phenotypically, fetal LTi cells and adult ILC3 populations can be distinguished on the basis of CCR6 expression, with LTi cells being of CCR6^hi^ phenotype and adult ILC3 populations of CCR6^low/−^ phenotype (14).

ILC3 are (arguably) the most interesting ILC in the intestine and are the most abundant ILC subset at mucosal tissues, particularly in the intestinal tract. In fact, ILC3 constitute the majority of ILC in the ileum (13, 49) and in the colon (19). Interestingly, it has been shown that the composition of the different ILC subsets correlates with localized IL7 expression. IL7 stimulation increases RORγt expression and this is a striking result, as the implication would be that IL7 does not just control ILC survival but can modulate which ILC subsets you have, i.e., the more IL7, the more ILC3.

In the healthy gut, ILC3 exhibit a range of cytokine dependent and cell surface receptor mediated mechanisms to apply homeostatic control of intestinal immunity. Indeed, one of the major functions of ILC3 is to maintain barrier integrity in an environment where a tug of war is constant between the enhancement and inhibition of immune responses against the microbiota (50).

Once they sense bacterial antigens, CX3CR1^+^ mononuclear cells and DC are major sources of IL23 or IL1β in the gut (Figure 1). In turn, these cytokines stimulate ILC3 to produce the effector cytokines IL22, GM-CSF, and IL17. The IL22 cytokine has a key role in homeostatic control and is secreted by Th17, Th22, and ILC3 (51–54). IL22 expression by ILC3 is also controlled by the aryl hydrocarbon receptor (Ahr), a ligand dependent transcription factor that is activated by xenobiotic and endogenous ligands (55). Ahr controls the development of adult, but not fetal RORγt^+^ ILC in mice, and RORγt^+^ ILC that lack Ahr were shown to produce less IL22 through both direct inhibition of *Il22* gene transcription and reduced responsiveness to IL23 (47, 48).

In the intestine, the IL22 dependent crosstalk between ILC3 and epithelial cells has been shown to influence the composition of the intestinal microbiota in mice, resulting in augmented host resistance to colonization with pathogenic organisms. The production of IL22 by ILC3 is critical for innate responses against intestinal bacterial pathogens such as *Citrobacter rodentium* (56), while their production of IL17 is central for antifungal responses against the opportunistic pathogen *Candida albicans* in the oral mucosa (57).

IL22 derived from ILC3 can also prevent bacterial translocation and keep opportunistic pathogens in check. Indeed, mice lacking IL22 or ILC3 show disrupted barrier integrity and hence cannot contain commensal bacteria resulting in translocation. ILC3 derived IL22 has been shown to protect mice against systemic dissemination of pathogenic bacteria following *Clostridium difficile* infection and infection-induced damage to the intestinal epithelium. ILC3 derived IL22 was required for the induction of complement factor C3 in the liver and intestine, which enhanced bacterial phagocytosis and allowed effective control of antigen load in peripheral organs (58). Additionally, IL22–IL22R interactions on intestinal epithelial cells (IEC) induce STAT3 phosphorylation and stimulate production and secretion of anti-microbial peptides and proteins such as β-defensins, RegIIIβ and RegIIIγ, calgranulins S100A8 and S100A9, and lipocalin-2 and mucins like Muc1, Muc3, Muc10, and Muc13 (59–63). In mice, it has been shown that suboptimal expression of anti-microbial factors by IEC results in systemic dissemination of commensal bacteria of the genus *Alcaligenes spp*, which is normally contained in GALT and Peyer’s patches. Peripheral *Alcaligenes spp*. dissemination was associated with low-grade systemic inflammation, which could be reversed by treating mice with recombinant IL22 (64).

IL22 binding to IL22R in IEC triggers a signaling cascade necessary for the production and maintenance of IL18 mRNA and pro-IL18 induction in epithelial cells (65). In addition, activation of the inflammasome in these cells leads to the downregulation of IL22 binding protein and hence increases the effects of IL22 (65, 66). Therefore, IL18 and IL22 production is tightly and mutually controlled contributing to the maintenance of gut homeostasis. When this delicate balance is altered under inflammatory conditions, intestinal pathology ensues. In mice, it has been shown that ILC3 responses are indirectly downregulated *via* IL25, which is secreted by microbiota-stimulated epithelial cells and likely acts *via* DC, which in turn limit ILC3 activation through a, so far, unknown mechanism (67).

In addition to being regulated by immune cell subsets such as DC (68), ILC3 have the capacity to regulate the activity of other immune cells *via* direct cell–cell interactions with important implications for gut homeostasis. Moreover, ILC3 respond to IL1β with the secretion of GM-CSF, a crucial regulator of mononuclear phagocytes, which induce intestinal Tregs. In fact, IL1β stimulation elicits production of pro-inflammatory cytokines by ILC3, including TNFα, IL6, and GM-CSF as well as regulating the expression of MHCII and co-stimulatory ligand expression in both *ex vivo* and *in vitro* generated mouse ILC3 (4). Hepworth et al. have demonstrated a requirement for class II transactivator, a master transcriptional regulator of MHCII, in driving MHCII expression by ILC3 (69). ILC3 are indeed capable of processing ingested protein antigens and load them onto MHCII for presentation to CD4 T cells, although the efficiency at which ILC were shown to do this was expectedly much lower than that of professional antigen presenting cells (5, 7).

ILC3 inhibit commensal-specific T cells *via* the MHCII receptor together with a withdrawal of IL2. Under steady-state conditions, IL2 and MHCII dependent interactions between intestinal ILC3 and effector CD4 T cells can inhibit CD4 T cell responses (5, 69). In humans, HLA-DR has been shown to be expressed on intestinal ILC3 as well as in ILC2. ILC3 have also been reported to limit commensal-specific CD4 T cell responses, through direct inhibitory interactions with CD4 T cells (5, 69) and through induction of tolerogenic RA and IL10 secreting intestinal DC and macrophages (70).

Data from our unit (Anna-Lena Schaupp et al.) have shown that ILC populations in human blood and the intestinal lamina propria express HLA-DR and co-stimulatory ligands, such as CD80, CD86, OX40, and PD-L1. In the colon, ILC3 were found to contain the highest proportion of HLA-DR expressing cells, with intermediate expression by ILC1 and lowest expression by ILC2. A similar distribution of HLA-DR expression among mouse ILC subsets in the small intestine has been reported with ILC3 populations containing a significantly higher percentage of HLA-DR^+^ cells compared with ILC2 or ILC1 (5).

Furthermore, ILC3 can also constrain commensal dissemination through effects on B cell responses. Thanks to their expression of B cell activating factors, such as BAFF, APRIL, and CD40L, as well as through lymphotoxin-dependent pathways, ILC3 promote the production of T cell-dependent and -independent IgA and IgG antibodies by mucosal and splenic B cell populations (71, 72). In turn, mucosal IgA and low-affinity systemic IgG antibodies contribute to the maintenance of mucosal homeostasis by regulating the composition, size, and anatomical containment of the intestinal commensal microbiota (73).

ILC3 express high levels of CD1d on their surface, therefore, they can acquire and present lipids to iNKT cells and this engagement stimulates them to produce IL22 (74). IL22 plays a central role in epithelial barrier function and tissue repair (75), and thus, ILC3 integration of signals obtained through CD1d, along with those received from cytokines in the local inflammatory milieu, such as IL23, may contribute to maintenance of homeostasis and to regulation of immune responses. By sensing the environment *via* the interaction microbial lipids-CD1d, ILC3 make an important contribution to the maintenance of barrier function.

The function of TLR on ILC3 is still an open question. TLR1, 2, 5, 6, 7, and 9 transcripts in human ILC3 have been shown to be expressed, albeit at low levels, however, only TLR2 engagement on ILC3 induces cytokine production in the presence of IL2, IL15, and IL23 (3). These results imply that ILC3 may have a role during *C. albicans* infection.

Another mechanism through which intestinal ILC3 have been shown to control commensal homeostasis is the regulation of IEC fucosylation (Figure 1). Commensal bacteria induce IEC fucosylation and epithelial fucose is used as a dietary carbohydrate. Fucosylated carbohydrates on IEC are thought to be important for the generation of an intestinal niche for commensal microbes (76). IL22 engagement with its receptor and secretion of lymphotoxin by ILC3 triggers fucosylation of epithelial cells (77). Indeed, IEC fucosylation is mediated by the fucosyltransferase enzymes Fut1 and Fut2 (78). ILC3 have been shown to induce intestinal epithelial Fut2 expression and fucosylation in mice. Abrogation of IEC fucosylation in mice lacking Fut2 has been linked to intestinal commensal dysbiosis and led to increased susceptibility to infection with the pathogens *Salmonella typhimurium* and *C. rodentium* (76). Thus, by regulating IEC anti-microbial functions, IL22 producing ILC3 plays an important role in preventing the systemic dissemination of microbial commensals.

## ILC in Intestinal Pathology

Recent evidence from murine and human studies indicates that ILC participate in the pathogenesis of chronic intestinal inflammation and colitis-associated cancer in patients with IBD. On the other hand, a protective role for ILC has emerged in murine models of graft versus host disease (GVHD), a major complication of allogeneic hematopoietic stem cell transplant (A-HSCT). These findings identify ILC as potential therapeutic target for patients’ treatment and suggest that modulation of ILC function and activity may be beneficial in pathological conditions.

### Inflammatory Bowel Disease

Inflammatory bowel disease is a chronic inflammatory disorder of the GI tract that encompasses two main forms, such as Crohn’s disease (CD) and ulcerative colitis (UC). In UC, inflammation is confined to the colonic mucosa while in CD it involves all layers of the gut wall and can affect any part of the GI tract, often leading to stricturing or fistulisation. Extra-intestinal manifestations of the disease are frequent with possible involvement of joints, skin, eyes, and kidneys. Moreover, the risk of developing other chronic immune disorders, such as psoriasis, ankylosing spondylitis, or primary sclerosing cholangitis (PSC), is also increased. Patients with long standing IBD colitis also have increased risk of colon cancer, and this risk is five times higher in patients with PSC-associated IBD (79). Patients are typically treated with immune suppressive treatment (corticosteroids, immunosuppressant agents, and biologic therapies, such as anti-TNFα or anti α4β7 antibodies), but surgery is often required during the course of the disease (80). The etiology of IBD remains unknown, but the available evidence suggests that IBD is caused by an abnormal immune response against the microorganisms of the intestinal flora in genetically susceptible individuals (81). Both dysregulated innate and adaptive immune pathways have been implicated in the pathogenesis of intestinal inflammation in these patients (82).

Early IBD research mainly focused on understanding the contribution of abnormal adaptive T cell responses to disease pathogenesis. CD has long been considered to be driven by an IL12 dependent Th1 response, while UC has been rather associated with a non-conventional Th2 response. However, an important role for IL23 driven Th17 responses in the pathogenesis of IBD has emerged by GWAS studies, and was confirmed in animal models and in translational studies in patients (83). Furthermore, advances arisen from genetic studies contributed to moving the focus of IBD pathogenesis on to mucosal innate immune responses. It became apparent that effector cytokines traditionally attributed to CD4 or CD8 T cells could also be secreted by unconventional innate lymphocytes, including ILC. This prompted an extensive research into the role of ILC in intestinal inflammation.

Buonocore et al. have shown that IL23 responsive ILC3 are required for induction of innate bacterial driven colitis in mice lacking B or T cells following infection with the pathogenic bacterium *Helicobacter hepaticus*. Colitis development was dependent on IL23 induced IL17 and IFNγ production by ILC3 that accumulated in the gut in response to IL1β. RORγt^+^ ILC3 were critical for colitis development, since intestinal inflammation was abrogated in mice that lack ILC3 (84). As previously mentioned, pathogenic IL17 production by intestinal ILC3 has also been shown to underlie the development of spontaneous colitis in mice lacking the type 1 transcription factor T-bet. In this model, ILC3 accumulate in the intestinal lamina propria and are activated in response to TNFα, IL23, and IL6 produced by activated DC (20).

Pearson et al. set out to investigate whether the movement of ILC within tissues contributed to immune and inflammatory responses (85). Using the anti-CD40 animal model of colitis, they observed ILC movement within cryptopatches. The authors concluded that by accumulating, clustering, and moving within the cryptopatches, ILC might initiate an inflammatory immune cascade resulting in intestinal inflammation. Interestingly, ILC appeared to wriggle their way directly through the tissue, possibly following a chemokine gradient.

Commensal dysbiosis secondary to ILC3 dysfunction can promote systemic inflammatory responses in mice. Outgrowth of commensal segmented filamentous bacteria, which are implicated in the development of pro-inflammatory Th17 cells (86), has been observed in mice lacking Ahr with lower levels of intestinal ILC3 derived IL22. Further reduction of ILC3 numbers and IL22 production in Ahr^−/−^Rorcγt^GFP/−^ mice promoted development of spontaneous, Th17-driven colitis, indicating that ILC3/IL22 mediated regulation of the commensal flora plays a role in mitigating the induction of potentially pathogenic Th17 responses to commensal antigens (48).

Furthermore, as already discussed, IL22 induces production of IL18 by IEC in mice and uncontrolled IL18 can amplify intestinal inflammation hence disrupting the mucosal barrier and creating a feedback loop of injury (65). Moreover, IL18 is up-regulated in intestinal lesions and peripheral blood in patients with CD (87–89), suggesting the presence of an altered IL22-IL18 regulation (Figure 1).

Interestingly, in both mice and humans, ILC3 were also found to secrete GM-CSF, which can recruit myeloid cells and further promote intestinal inflammation (85). In the same study, it was shown that ILC3 move within the intestinal tissue when activated. These two mechanisms can contribute to the induction and progression of inflammation throughout the gut.

In intestinal samples from IBD patients, we observed accumulation of Lin^−^CD56^−^CD127^+^ ILC in the inflamed ileum and colon of patients with CD, but not UC. These cells were able to respond to IL23 *in vitro* and they expressed IL17 and IFNγ (90). More, recently, we also found increased ILC frequencies in patients with PSC-associated IBD, but again no accumulation was observed in UC patients (91). Follow-up studies have confirmed that ILC contribute to inflammation in CD and suggested that IFNγ secreting ILC1 are especially increased in the intestinal lesions in these patients. Two populations of ILC1 have been described in the human intestine. Bernink et al. identified a population of CD127^+^NKp44^−^c-Kit^−^ cells that express high levels of T-bet and IFNγ compared to c-Kit^+^ ILC3 (13). Similarly to NK cells, these cells respond to IL12 and IL18 stimulation *in vitro* with induction of IFNγ, however, they do not express granzyme B or perforin, which are classically associated to NK cell cytotoxic ability. Another population of IFNγ secreting intraepithelial ILC1, which are CD127^−/low^ and express the epithelial homing integrin CD103, was identified in the human gut (92). Like CD127^+^ ILC1 and CD127^−/low^CD103^+^ ILC1 are T-bet^+^, but they express the transcription factor Eomes and perforin and granzyme B granules, similarly to conventional cytotoxic NK cells. IFNγ secreting CD127^+^NKp44^−^C-Kit^−^ ILC1 were found to accumulate in the inflamed intestine of patients with CD (13). The frequency of intraepithelial CD127^−/low^CD103^+^ ILC1 was also found to be increased in the inflamed tissue of patients with CD (92). These data are in agreement with our first observation of increased frequencies of ILC in the inflamed lamina propria of patients with CD and may reflect ILC functional flexibility and their exposure to high levels of IL12 in the mucosa (90, 93) (Figure 1).

A role for ILC2 in pathologic intestinal inflammation has also been suggested by results in the oxazolone induced model of colitis, where ILC2 participate in intestinal inflammation in an IL25-dependent manner (94). Interestingly, increased frequencies of ILC2, which are able to secrete IL13 and IFNγ, have been detected in intestinal samples from CD patients (15). In the same study, stimulation of ILC2 with IL12 *in vitro* induced the expression of T-bet and IFNγ. IL12 is classically increased in the mucosa of patients with CD (93) and the finding of increased frequency of IFNγ secreting ILC2 may, therefore, reflect the functional plasticity of ILC depending on the mucosal microenvironment. CRTH2^+^ ILC2 were found to accumulate in esophageal tissues of patients with the allergic disease eosinophilic esophagitis (95), in nasal polyps of patients with chronic rhinosinusitis (36), an allergic condition characterized by high levels of IL5 and IL13, and in the inflamed skin of patients with atopic dermatitis (96), suggesting they can contribute to type 2 cell-mediated disease. In these allergic conditions, CRTH2^+^ ILC2 can be activated by mast cell released PGD2, up-regulate IL33R and IL25R and secrete IL4, IL5, and IL13 leading to the amplification of the type 2 response (30). On the other hand, while IL13 induction has been associated to mucosal inflammation in patients with UC (97, 98), there are no reports of increased ILC2 frequency in UC to date. Very low frequencies of ILC2 are found in the human colon and they may not represent major players in the colonic immune response, at least in the absence of parasitic infections (19). Interestingly, frequencies of CD127^+^ ILC were similar between UC patients and controls in our studies (90, 91), suggesting these cells may not be involved in intestinal inflammation in these patients.

### Colorectal Cancer

Inflammation is one of the 10 hallmarks of cancer (99). The immune system can profoundly influence carcinogenesis with both favorable anti-tumorigenic effects and detrimental pro-tumorigenic activities. Despite the protective role of mucosal inflammation to limit damage and promote tissue repair, a dysregulated, chronic and extensive intestinal inflammation is regarded as a promoter of carcinogenesis. The exact mechanisms remain unknown, but inflammatory cytokines can act on IEC to promote many processes involved in neoplastic transformation, including proliferation, inhibition of apoptosis, invasion, angiogenesis, epithelial to mesenchymal transition, and metastasis (100).

Evidence from animal models suggests that ILC and especially IL23 responsive ILC3 may contribute to intestinal carcinogenesis. In particular, Kirchberger et al. identified a role for IL23 driven IL22 producing ILC in bacteria-induced colitis-associated cancer in T and B cell deficient mice infected with *H. hepaticus* long-term or co-treated with 2-azoxymethane (a cancer inducing agent) (101). In this setting, IL22 producing CD4^−^Nkp46^−^Thy-1^+^ ILC accumulate at the expense of Nkp46^+^ and CD4^+^ ILC populations. Depletion of Thy-1^+^ ILC or IL22 blockade can reverse established invasive carcinoma and inflammation. Importantly, IL22^+^ T cells and non-T cells are present in human colorectal cancer, and *IL22* gene expression is increased in cancer tissue relative to matched adjacent normal tissue in these patients. In another model, transgenic expression of IL23 was shown to drive the *de novo* development of duodenal adenomatous tumors that was dependent on IL17 secreting Thy-1^+^ Sca-1^+^ ILC (102). However, one must be careful to translate results from T cell depleted animals into clinical settings, especially in light of the beneficial effects of IL22 in promoting barrier protection and epithelial regeneration. It is evident that IL22 can act as a “double edged sword” with both protective and pathogenic activities depending on the context.

Patients with IBD are at increased risk of developing intestinal cancer. This risk is further increased in patients co-diagnosed with PSC, a chronic progressive disorder of the hepato-biliary system, and strongly associated to IBD. PSC is characterized by inflammation, fibrosis, and stricturing of intrahepatic and extrahepatic bile ducts, leading to liver cirrhosis (103). Interestingly, patients with UC and PSC have a fivefold increased risk to develop colon cancer than those with UC alone (104). Patients with PSC are also at increased risk of other malignancies, including cholangiocarcinoma, gall-bladder adenoma, dysplasia, and carcinoma (105).

Our data show that secretion of Th17-cytokines from colonic T cells did not differ between patients with PSC–IBD, patients with UC and healthy controls, while we observed enhanced Th1 responses in PSC–IBD. On the other hand, ILC accumulate in the colon of PSC–IBD patients suggesting that ILC may play a role in intestinal inflammation and increased cancer risk in PSC and IBD (91). However, further studies are needed to evaluate the functional activity of ILC in these patients, and whether they contribute to pro-carcinogenic responses, including cytokine secretion and/or cell–cell interaction, that can promote epithelial transformation in PSC–IBD.

### Graft Versus Host Disease

Allogeneic hematopoietic stem cell transplant is a potentially curative treatment for patients with leukemia and lymphoid malignancies (106). However, A-HSCT is frequently complicated by potentially fatal GVHD (107), caused by allo-reactive donor T cells that are activated against recipient antigens and lead to severe tissue damage, most frequently involving the GI tract, liver, and skin, where ILC are particularly represented.

Murine studies showed that IL23 responsive ILC3 secrete IL22 after bone marrow transplant, but are decreased in mice with acute GVHD. The reduction of ILC3 is associated with decreased secretion of IL22, loss of intestinal stem cells, impaired epithelial barrier function, and increased intestinal inflammation (108). A recent study showed that ILC2 are highly reduced after radiation and chemotherapy before A-HSCT in mice, and only limited repopulation with donor bone marrow derived ILC2 is observed in the GI tract. Interestingly, infusion of IL33–activated donor ILC2 significantly reduced the severity of GI GVHD in two separate murine models and this effect was dependent on the recruitment of donor myeloid-derived suppressor cells. Furthermore, ILC2 treatment of recipients with established GVHD reduced disease severity and increased survival (109).

Accordingly, ILC are depleted by pre-conditioning therapy in patients with acute leukemia and the reconstitution of ILC1, ILC2, and NKp44^−^ ILC3 is slow compared with neutrophils and monocytes. On the other hand, NKp44^+^ ILC3 that are not present in the peripheral blood of healthy individuals, appear after conditioning and A-HSCT. A significant proportion of NKp44^+^ ILC3 expresses the gut-homing and skin-homing receptors, cutaneous lymphocyte-associated antigen and CCR10. The frequency of activated NKp44^+^ ILC3 expressing skin- and gut-homing receptors were significantly higher in patients who did not develop therapy induced mucositis and acute or chronic GVHD. These data suggest that NKp44^+^ ILC3, similarly to their murine counterpart, are involved in intestinal homeostasis and tissue repair contributing to GVHD prevention possibly through the secretion of IL22 (110).

On the other hand, ILC were shown to be dispensable in patients with severe combined immune deficiency that received A-HSCT. In these patients, while T cell and B cell reconstitution was obtained after A-HSCT, ILC were still deficient after transplant. However, no increased risk of infections or other disease was observed in the medium-long term in these subjects (111).

Besides their protective role in maintaining intestinal homeostasis, ILC have been shown to participate in chronic intestinal inflammation in both animal models and human diseases. In the presence of pro-inflammatory mediators in the microenvironment a shift from protective ILC populations toward pro-inflammatory and possibly pathogenic ILC subsets may occur. While IL22 mediated induction of epithelial proliferation and tissue repair may play a protective role in intestinal inflammation in GVHD, chronic exposure to IL22 may lead to acquisition of epithelial stemness and neoplastic transformation contributing to the increased risk of colon cancer that is associated to longstanding colitis.

## Future Perspectives and Therapeutic Applications

The identification of ILC as a heterogeneous group of innate lymphocytes particularly abundant at mucosal sites has prompted both animal and human studies aimed to evaluate their role in intestinal pathophysiology. They are present at low frequencies, compared to other lymphocytic or myeloid populations, however, results from murine studies have highlighted their important contribution to maintaining intestinal homeostasis and to promoting chronic intestinal inflammation. As discussed, ILC can exert protective activities in the gut, through regulation of the microbial niche and contribution to epithelial and tissue repair. On the other hand, they can induce inflammation through the production of inflammatory cytokines and the regulation of innate and adaptive immune responses. It is becoming apparent that ILC can exert their function, not only through the secretion of soluble mediators, but also through their interaction with other cells, such as immune cells, including myeloid and T cell populations, and non-immune cells, such as epithelial cells and stromal cells, as suggested by the role of LTi-stromal cell crosstalk during lymphoid tissue embryogenesis. Targeting ILC activities, through cytokine blocking or modulation of ILC activation and their interaction with other cells, may result in beneficial effects in order to maintain homeostasis and prevent or treat inflammation. However, due to the lack of ILC specific markers, mediators or functions, at present any therapeutic approach will also have effects on other immune cells, and particularly on T cell mediated responses.

IL22 is emerging as a central mediator in intestinal homeostasis and tissue repair (75), while IL17 and IFNγ preferentially secreted by ILC3 and ILC1 have been associated to more pathogenic effects. A differential accumulation of IL22 secreting and IFNγ− and IL17A-secreting ILC has been found in patients with IBD and particularly in CD. Our own data suggest that ILC populations that respond to IL23 stimulation with secretion of IL17A and IFNγ accumulate in the inflamed intestine of patients with CD, but not in UC. Further studies have confirmed accumulation of IFNγ secreting ILC populations in both the lamina propria and the epithelium in CD (13, 90). Moreover, a reduction in the frequency of IL22 secreting ILC3, associated to IFNγ secreting ILC accumulation, has been described in CD (112, 113). Interestingly, no study to date has described accumulation of ILC in the gut of patients with UC and our studies may suggest they are not implicated in UC inflammation, even if larger studies are needed. On the contrary accumulation of ILC is present in patients with PSC–IBD, who classically exhibit mild colitis but significantly increased risk of colon cancer compared to other patients with IBD (91).

ILC1 secreted IFNγ may contribute to propagation of inflammation and tissue damage in CD. Both Th1 and ILC1 responses are increased in CD with higher levels of IFNγ expressed in the intestinal mucosa of these patients. However, Fontolizumab, a humanized murine anti-IFNγ antibody, did not show efficacy in clinical trials, even if some concerns about the design of these studies have been raised and Fontolizumab may induce some partial benefit in CD (114).

ILC3 derived IL17 promotes the recruitment of inflammatory neutrophils by inducing the release of chemokines from epithelial and endothelial cells. In this context, IL17 promotes epithelial barrier function (115). However, IL17 also presents cytokine functional dichotomy, and in the presence of IL23, IL17 and pathogenic Th17 cells have been implicated in intestinal inflammation and tissue damage (116). Hence, IL23 dependent IL17 production might be counter-indicative. Based on the results of genetic studies and immunological studies models and human tissue, blocking Th17- and unconventional lymphocyte-secreted IL17 seemed a reasonable therapeutic approach for patient with IBD. However, specific targeting of IL17 with Secukinumab, a human anti-IL17A monoclonal antibody, led to disappointing results in a multicentre phase IIa study (117). Secukinumab was not only ineffective in CD patients, but also actually resulted in disease exacerbation, likely interfering with the more protective IL17 functions or resulting in increased Th1 and IFNγ-mediated responses. The results of this study highlighted the complexity of the IL23/IL17 axis, with IL17 being only one of the many IL23 induced mediators with often redundant and dichotomic effects (118).

It is conceivable that insufficient levels of intestinal ILC derived IL22 in CD patients, resulting in impaired epithelial regeneration, innate anti-microbial defense and containment of commensal microbes, may contribute to disease pathogenesis. Selective enhancement of IL22 production by ILC may have protective effects in these patients, although this strategy would have to be carefully balanced against a potentially increased risk of colorectal cancer. In fact, chronic IL22 exposure promotes intestinal epithelial proliferation and may result in increased risk of colitis-associated cancer as suggested by murine studies (101). Targeting of IL22 or more specifically IL22 secreting ILC may be beneficial in the prevention or treatment of longstanding IBD-associated increase risk of colon cancer.

Studies in murine models also suggested that blocking ILC3 secreted GM-CSF or altering ILC3 movement or activity might help reduce intestinal inflammation (85). However, blocking GM-CSF activity could also result in the inhibition of important protective mechanisms (119, 120). Furthermore, anti-GM-CSF autoantibodies were found to be increased in IBD (121) and recombinant GM-CSF (Sargramostim) showed some efficacy in patients with CD (122). However, these results were not confirmed in further studies (123). In any case, clinical trials are underway to investigate the use of anti-GM-CSF in other inflammatory conditions, such as rheumatoid arthritis or multiple sclerosis and these studies could offer insight into whether this approach could have beneficial effect on intestinal inflammation in patients with concomitant IBD.

ILC3 might well be divided into several subsets and an understanding of the different functions of each one might help us in the design of specific therapies according to the specific roles of ILC3 in intestinal inflammation and homeostasis. A crucial open question of ILC biology is how these rare cells impact homeostatic or, in the case of IBD, pathogenic immune responses in the presence of a functional adaptive immune system. This is evidenced by the existence of functionally discrete ILC3 subsets, including those that present antigen and inhibit microbiota-directed T cell immunity and those that promote anti-microbial T cell responses *via* IL22 production.

Elevated levels of pro-inflammatory cytokines, such as IL12, IL23, or IL1β, in the intestine of patients with IBD may be responsible for the acquisition of pathogenic ILC phenotypes. IL12 and IL23, can be targeted by Ustekinumab and Briakinumab, monoclonal antibodies against their shared p40 subunit. This strategy has proven to be effective for patients with CD (124–126) and it remains to be elucidated how ILC and T cell populations are affected by IL23 and IL12 blockade. Interestingly, the efficacy of anti-IL12/IL23 treatment in CD is not comparable to the striking results obtained in patients with psoriasis (127, 128) highlighting the complexity of the immune mechanisms involved in intestinal inflammation in these patients (118). More recently Risankizumab, a monoclonal antibody against the IL23 specific subunit p19, showed higher efficacy than placebo at inducing clinical remission in CD patients in a phase 2 study (129).

It is conceivable that future therapeutic strategies could include interfering with ILC interaction with immune and non-immune cells. As discussed, ILC3 inhibit commensal-specific T cells *via* the MHCII receptor together with a withdrawal of IL2 (5, 69). However, in IBD an increase in the availability of IL2 (130) due to widespread immune activation could mean that IL2 uptake by ILC may no longer be a limiting factor for effector CD4 T cells. In this context, T cell-derived IL2 in conjunction with other pro-inflammatory cytokines (IL1β, IL6, IL12, and IL23) could furthermore drive inflammatory ILC3 activation. ILC3 derived effector cytokines (IL17A, IFNγ, TNFα, and GM-CSF) could then feedback to potentiate pathogenic effector CD4 T cell responses, either directly or indirectly *via* antigen presenting cells.

Taken together, a better understanding of the regulation of cytokine expression by ILC and their interaction with other immune and non-immune cells will help to develop new strategies to treat inflammatory diseases in humans.

## Author Contributions

AG and CVA-C contributed equally to this work.

## Conflict of Interest Statement

The authors declare that the research was conducted in the absence of any commercial or financial relationships that could be construed as a potential conflict of interest.
